# On the Filling and Treatment of the Permanent Molar Teeth between the Ages of Six and Fourteen Years

**Published:** 1882-10

**Authors:** Gascoigne Palmer

**Affiliations:** England


### On the Filling and Treatment of the Permanent Molar Teeth between the Ages of Six and Fourteen Years.

BY GASCOIGNE PALMER, L.D.S., ENGLAND.*

Mr. President and Gentlemen:—The subject I have chosen is of such exceeding interest, of such vital importance, to all who are really interested in Conservative Dentistry, that it seems to me that I could more easily write a book on it, than a paper. I am, moreover, far from being an adept in the art of putting a great amount of matter into very few words, but as there are seven other papers to be read this afternoon I will try to make this one as short as possible.

First of all, who is there here present who, on looking into the mouth of a new patient, say twenty years of age, has not very often been, well, almost scared by the small amount of mastication performed by the first and second molars of the second set of teeth. Now, those eight teeth ought, if they were all good, to do certainly three-fourths of the grinding of the food and one-half of the process of thoroughly salivating it, if I may be allowed the expression, a very necessary process to the perfect digestion of the food. Well, what do you often find? One, two, three, four, five, six, seven, eight, all gone; the wisdom teeth well cut and hanging over towards the front of the mouth, like anything rather than a molar tooth, offering little or no masticating surface; the alveolar process of the jaw absorbed, the bicuspids standing with the cementum as much bared as though the patient was fifty; the incisors showing most unmistakable signs of being used for what they were not intended, and the bicuspids of doing more than they were intended to do.

Take another case: All the molars are present, but what a

* Read at the Annual General Meeting of the Western Branch, held at Cheltenham, on the 5th inst.

condition the mouth is in. All the molars encrusted with tartar, breath fetid, tongue foul, patient nervous and complains that "these teeth, you know, they are so tender I can't bite on them, and they sometimes ache very badly. Would it not be better to have them all out and have new ones, for they have been stopped so often and I don't believe they will ever get better." Such is the wail of many a disheartened patient. Now when we come to think that in both cases the patient has been in the habit of going to respectable dentists, and having done whatever has been recommended—how is it, I ask, that we see so many of such cases? I know of hundreds of such instances with various differences; I have simply stated two extremes. Why, because there is no regular systematic way of treating molars when the pulps are large, often small hair-like fibres taking abnormal positions, the dentine so elastic that a solid plug of gold, when put in, will press down on the too sensitive pulp—the dentinal tubes so moist that thermal changes are instantly felt whether the tooth is unstopped or stopped with metal; in fact because there is a great deal too much of "Here's a hole, put something in it," in the minds of many an overworked dental surgeon; and "Well, I am sure I do the best I can," for an excuse. And so thousands of teeth are lost for all practical purpose, even if they are not taken out. How then is this sad loss of teeth to be avoided? First of all by not being in a hurry; don't do more than examine a mouth unless an appointment has been made. If you make a mistake in a hurried opinion, on another occasion you can correct it; but if you make a mistake in operating on a young molar tooth, I do not believe you can correct it; and I do believe that in many instances where, through a mistake, serious periostitis takes place in a young tooth, the best thing is to extract the tooth, rather than run the risk of endangering all the teeth on that side of the mouth by the roughening of the enamel from want of friction in masticating.

Now, sir, having laid before this meeting, as briefly as possible, the views which have induced me to read this paper, I will present to your notice three forms in which decay of the permanent molars present themselves to our notice, and I must ask you to bear in mind that my remarks apply to molars in the mouths of patients between six and fourteen years of age.

In order to simplify things as much as may be, we will say that all the cavities shall be coronal ones, and none of them ever treated before.

Class 1. Shall consist of teeth that have a hole in them, but which have never even given so much as one single twinge of pain.

Class 2. Shall consist of those that have given twinges of pain and which are affected by heat or cold.

Class 3. Shall consist of those that have ached or are aching.

Taking the last first, let me say that no great amount of success will attend the treatment of these teeth if the patient be under fourteen, still less if under eight; they are either dead teeth, or in a fair way of becoming so, and with large pulp canals and apical foramina, the chance is that the ordinary applications for such teeth, the use of the nerve extractor, Donaldson's bristles, etc., etc., will set up some inflammation of the alveolar process, and in the end the tooth will have to come out, remain without a stopping in it, or be plugged with a risodontrophy hole in it for the exit of discharge, and so we will say no more on that class.

Classes 1 and 2 may be almost considered together, except as to treatment and plugging, and then class 2 only requires more delicate manipulation and much greater care in the selection of the material used for filling.

Now, the thing that strikes me as of the first importance in classes 1 and 2 is thoroughly to diagnose the fact, to our own most complete satisfaction, that they do not belong to class 3; in other words, that there is no pulp exposure and no inflammation, not even a little bit of the pulp itself. Being quite sure of our way so far, the next thing to do is to prepare the cavity. I find as a rule the best way to do this is to cut away with an excavator the carious bone immediately under the enamel—not to go down to the floor of the cavity—and then break down the walls with an enamel chisel; or, if the patient be a very sensitive one, grind out with a cone-pointed corundum with the engine. Then before going further, let me say this, if we are sure that there is no necessity to "destroy the nerve," as the saying is, the use of arsenic for the purpose of getting rid of sensitiveness of dentine is simply unjustifiable. There is no way in which any one can court failure more certainly than by using arsenic in such a case. Although what I have just read is a slight digression, I shall not apologise for making it, for I fear that the practice of using arsenic for getting rid of sensitiveness in dentine is far too common; and, if there is anything I am glad to have had the opportunity of reading this paper for, it is for the purpose of putting my foot down on the reckless and indiscriminate way in which arsenic is used.—(Amen.—*Ed. Reg.*)

But to resume. Having got rid of all undermined enamel, and, in the most careful manner, of every atom of the softened dentine immediately contiguous, put on the dam, place the ejector in the mouth, and dry your cavity thoroughly; wait for a minute or two to see that all is as water-tight as you wish, then soak the softened dentine in Fletcher's ether and copal varnish (a most valuable preparation, worth all the other things he ever brought out, except his stoves), and fill—well, with what? I use Ash's rock cement, and, if it is used really dry, I know nothing to approach it. But at all events don't use metal; the thermal changes are too risky to the preservation of the pulp in patients under fourteen years of age.

So much for classes 1 and 2 taken together. But supposing we take class 2 as wanting more care, which in my opinion it does, than class 1, where is the extra care to be developed? In this: in taking greater care that the irritating properties of the preparations of zinc do not penetrate through the softened dentine overlying the pulp, and produce acute pulpitis. I will describe to you a mode I have used for the last four years with very great success—in fact I do not know of a single failure, though, in this peripatetic town, where people are always going and coming, I dare say there have been some.

Having got the dam on to my most complete satisfaction—and that means a certainty of no moisture finding its way to the cavity under treatment—I vary my manner of filling in this way: Having soaked the softened dentine in Fletcher's copal and ether varnish, I take a piece of gutta percha tissue and force it down with small pieces of oiled Japanese bibulous paper until it thoroughly adheres to the softened dentine, which it readily does by reason of its being saturated with the copal and ether varnish, the ether dissolving a certain portion, no doubt, of the gutta percha tissue. In this way I have filled many teeth with oxychloride of zinc without the slightest after pain ; and I am sure that if any of you try this plan of mine, you will find out how many teeth may be retained alive in the mouths of young people that would otherwise most certainly "spontaneously" die, as it is supposed, and as I do not believe. True spontaneous death of the pulp is to my mind very rare.

Now, in conclusion, let me most emphatically say that I do not think any metal or any amalgam is as good for filling molar teeth in children under fourteen as a good oxychloride of zinc stopping; and although small enamel fissures may be filled with gold without endangering the life of the tooth, still, at the same time, so predisposed are these molars to decay, that I think the probability is that the oxychloride would serve the purpose just as well, and, in these days of charging by time, cost a great deal less in putting in.

Sir and gentlemen, I thank you very much for your kindness in listening to my paper, and I sincerely hope that its contents may be of some use, if not now, some day, in preserving those useful articles, the first and second permanent molars.—*Journal of the British Dental Association.*

